# Neurocognitive Dysfunction in Systemic Lupus Erythematosus: Association with Antiphospholipid Antibodies, Disease Activity and Chronic Damage

**DOI:** 10.1371/journal.pone.0033824

**Published:** 2012-03-26

**Authors:** Fabrizio Conti, Cristiano Alessandri, Carlo Perricone, Rossana Scrivo, Soheila Rezai, Fulvia Ceccarelli, Francesca Romana Spinelli, Elena Ortona, Massimo Marianetti, Concetta Mina, Guido Valesini

**Affiliations:** 1 Lupus Clinic, Reumatologia, Dipartimento di Medicina Interna e Specialità Mediche, Sapienza Università di Roma, Rome, Italy; 2 Dipartimento di Biologia Cellulare e Neuroscienze, Istituto Superiore di Sanità, Rome, Italy; 3 Department of Neurology and ORL, Sapienza Università di Roma, Rome, Italy; Federal University of Rio de Janeiro, Brazil

## Abstract

**Introduction:**

Systemic lupus erythematosus (SLE) is characterized by frequent neuropsychiatric involvement, which includes cognitive impairment (CI). We aimed at assessing CI in a cohort of Italian SLE patients by using a wide range of neurocognitive tests specifically designed to evaluate the fronto-subcortical dysfunction. Furthermore, we aimed at testing whether CI in SLE is associated with serum autoantibodies, disease activity and chronic damage.

**Methods:**

Fifty-eight consecutive patients were enrolled. Study protocol included data collection, evaluation of serum levels of ANA, anti-dsDNA, anti-cardiolipin, anti-β_2_-glycoprotein I, anti-P ribosomal, anti-endothelial cell, and anti-Nedd5 antibodies. SLEDAI-2000 and SLICC were used to assess disease activity and chronic damage. Patients were administered a test battery specifically designed to detect fronto-subcortical dysfunction across five domains: memory, attention, abstract reasoning, executive function and visuospatial function. For each patient, the raw scores from each test were compared with published norms, then transformed into Z scores (deviation from normal mean), and finally summed in the Global Cognitive Dysfunction score (GCDs).

**Results:**

Nineteen percent of patients had mild GCDs impairment (GCDs 2–3), 7% moderate (GCDs 4–5) and 5% severe (GCDs≥6). The visuospatial domain was the most compromised (MDZs = −0.89±1.23). Anti-cardiolipin IgM levels were associated with visuospatial domain impairment (r = 0.331, P = 0.005). SLEDAI correlated with GCDs, and attentional and executive domains; SLICC correlated with GCDs, and with visuospatial and attentional domains impairment.

**Conclusions:**

Anti-phospholipids, disease activity, and chronic damage are associated with cognitive dysfunction in SLE. The use of a wide spectrum of tests allowed for a better selection of the relevant factors involved in SLE cognitive dysfunction, and standardized neuropsychological testing methods should be used for routine assessment of SLE patients.

## Introduction

Systemic lupus erythematosus (SLE) is an autoimmune disease characterized by frequent neuropsychiatric involvement that could be found up to 80% of patients [1]–[7]. Neuropsychiatric SLE (NPSLE) includes a wide range of neurological and psychiatric manifestations as well as cognitive impairment (CI). In 1999, the American College of Rheumatology (ACR) proposed a standard nomenclature for NPSLE with case definitions for nineteen neuropsychiatric syndromes associated with SLE [1]. So far, there is no reliable diagnostic test, which makes the diagnosis of NPSLE difficult. Manifestations of NPSLE vary in severity, ranging from mild headache to life-threatening coma [8]. Advances in research methodologies and the introduction of neuropsychological methods have improved clinicians' ability to identify CI in both pediatric and adult SLE patients [9].

CI in SLE is characterized by deficits in attention, learning and recall, verbal and nonverbal fluency, language, visuospatial skills, executive functions and motor dexterity and is probably due to a damage of fronto-subcortical circuits [4]–[6]. The prevalence of CI in SLE patients was found to be comprised between 3–80% of patients [10]–[14].

This apparent discrepancy is mainly due to the different tests that were administered in these studies, and by the abovementioned only recent application of a specific nomenclature for CI in SLE patients. Petri et al. in 2008 found that after adjusting for age, gender, ethnicity, and education, SLE patients score significantly lower than controls on measures of cognitive efficiency requiring sustained attention/vigilance, visuospatial span of attention/working memory, and simple reaction time [15]. Nonetheless, it was showed that CI in children and adolescents with SLE can affect intelligence, academic achievement, arithmetic, reading comprehension, learning, visual memory and complex problem solving ability [16].

The pathogenesis of NPSLE has been attributed to autoantibody-mediated neuronal dysfunction, vasculopathy, and coagulopathy [17]–[19]. It has been suggested that several autoantibody specificities may play a role in the pathogenesis of NPSLE [reviewed in 20]. Among others, a potential pathogenic relevance has been attributed to anti-neuronal, anti-P ribosomal proteins, anti-phospholipids (aPL), and human N-methyl-D-aspartate (NMDA) receptor types NR2a or NR2b (anti-NR2) antibodies [20]–[29]. Recently, we demonstrated an association between the presence of anti-endothelial-cell antibodies (AECAs) and anti-Nedd5 C-ter antibodies with psychiatric manifestations, such as psychosis and depression, in SLE [24], [25]. In 1999, the ACR Ad Hoc Committee on Neuropsychiatric Lupus nomenclature proposed a brief research battery of neurocognitive tests to quantify cognitive dysfunction in SLE [1]. In 2007 the response criteria for neurocognitive impairment in SLE clinical trials were proposed, and the combination of the ACR neuropsychological battery with the Cognitive Symptoms Inventory (CSI) [30] was suggested to evaluate cognitive function [1], [31]. The objective of the present study was to assess cognitive dysfunction in a cohort of Italian SLE patients by using a wide range of neurocognitive tests, including those from the ACR and the CSI, specifically designed to evaluate the fronto-subcortical dysfunction typical of NPSLE. Furthermore, we aimed at testing whether CI in NPSLE was associated with serum autoantibodies, including anti-dsDNA, aPL, AECA, anti-Nedd5, and anti-P ribosomal, and with disease activity and chronic damage.

## Materials and Methods

Fifty-eight consecutive patients ≥16 years of age affected with SLE, as diagnosed according to the ACR revised criteria [32], were enrolled in this cross-sectional study at the Lupus Clinic, Sapienza University of Rome. Written informed consent was obtained from each patient and the ethic committee of Sapienza Università di Roma approved the study design.

Study protocol included complete physical examination and blood drawing. The clinical and laboratory data were collected in a standardized computerized electronically-filled form including demographics, past medical history with date of diagnosis, co-morbidities, and previous and concomitant treatments. Clinical activity was assessed using the SLE Disease Activity Index (SLEDAI) [33], while chronic damage was evaluated using the Systemic Lupus International Collaborative Clinics/American College of Rheumatology (SLICC/ACR) Damage Index [34], [35]. Each subject underwent peripheral blood sample collection. The sera recovered were then stored at −20°C until assayed.

Methods of assessment of AECA and anti-Nedd5 antibodies have been previously described [24], [25]. In brief, human umbilical-vein endothelial cells were isolated by collagenase perfusion from normal-term umbilical cord veins [36] and were cultured in M19 medium (Sigma Chemical Co, St. Louis, MO, USA) supplemented with 20% FCS. These cells (third to fourth passage) were used to detect AECA of IgG isotype using a cell-surface ELISA on living cells allowed to grow to confluence in microtiter plates. After three washes with Hank's balanced salt solution (HBSS), nonspecific binding sites were blocked for 2 hours at room temperature with 3% bovine serum albumin (BSA)/HBSS. After two washes with HBSS, the wells were incubated in duplicate with 100 µl of the sera diluted 1∶50 in HBSS for 2 hours at room temperature. After three washes with HBSS, the bound antibodies were detected with alkaline-phosphatase-conjugated goat antibodies antihuman IgG (Sigma), using 1 mg/ml p-nitrophenylphosphate. Optical density (OD) was measured at 405 nm wavelength and AECA were expressed as a binding index (BI), equal to 100×(S-A)/(B-A), where S is the OD of the sample tested, A is the OD of a negative control, and B that of a positive reference serum. AECA were considered positive when the BI was higher than the cutoff value (mean+2 standard deviations (SD) of 66 healthy controls) corresponding to 50% of a positive reference serum from an SLE patient [36].

ELISA for specific total anti-Nedd5 IgG was developed as previously described [25]. Briefly, polystyrene plates (Dynex, Berlin, Germany) were coated with Nedd5 C-ter 0.5 µg/well in 0.05 µM NaHCO3 buffer, pH 9.5. Coated plates were incubated overnight at 4°C and then washed three times with PBS containing 0.05% Tween-20 in an automated washer (Wellwash 4, Labsystem, Turku, Finland). Plates were blocked with PBS Tween containing 3% gelatin (Bio-Rad), 100 µl/well, for 1 hour at room temperature and washed as previously described [25]. Human sera were diluted in PBS Tween-20 and 1% gelatin (1∶100 for total IgG) and pipetted onto plates at 100 µl per well. Plates were incubated for 1 hour at 20°C and washed as described. Peroxidase-conjugated goat antihuman IgG (Bio-Rad) was diluted 1∶3000 in the same buffer. These dilutions were used as second antibodies and incubated (100 µl/well) for 1 hour at 20°C. o-Phenylenediamine dihydrochloride (Sigma) was used as a substrate and absorbance was measured at 490 nm. Means+2 SD of the absorbance reading of the 66 healthy controls were considered the cutoff levels for positive reactions. All assays were performed in quadruplicate. Data were presented as the mean OD corrected for background (wells without coated antigen). The results of unknown samples on the plate were accepted if internal controls (two serum samples, one positive and one negative) had an absorbance reading within mean ±10% of previous readings. To inhibit specific IgG, the sera from three patients with SLE, IgG anti-Nedd5 positive, were diluted 1∶50 in PBS-Tween and were incubated overnight at room temperature in 10 µg/ml of Nedd5 C-ter according to the method reported by Huang and colleagues [25]. As a negative control, the sera were pre-incubated with 40 µg/ml of BSA.

Anti-cardiolipin (anti-CL), anti-P ribosomal proteins (P0, P1), and anti-β_2_ glycoprotein I (GPI) ELISA kits were obtained from Diamedix (Miami, FL, USA, 30). Anti-CL of IgG and/or IgM isotype as well as anti-β_2_GPI of IgG and/or IgM isotype in serum were expressed as immunoglobulin G phospholipid (GPL) or immunoglobulin M phospholipid (MPL) units using international reference material and considered positive at a titer >20 GPL or MPL. Anti-dsDNA antibodies were performed in indirect immunofluorescence in accordance with the manufacturer's instructions. All assays were performed in duplicate. A positive control and several normal human sera were run in the same assay to confirm the specificity of the results. Levels of aPL, anti-P ribosomal, AECA and anti-Nedd5 were categorized as absent (Z score<1), low (Z score between 1 and 2), or high (Z score>2) after comparison with healthy controls, as previously described [26].

All patients underwent an extensive cognitive-behavioral neuropsychological assessment. Neurocognitive assessment was performed during a 1-hour interview and included standardized testing for 5 domains: memory, attention, abstract reasoning, executive and visuospatial functions. This assessment included those tests from the ACR and the CSI standardized in an Italian population, and was specifically designed to detect the fronto-subcortical dysfunction typical of SLE.

All the patients were administered a Minnesota Multiphasic Personality Inventory (MMPI) to exclude that cognitive dysfunction could be related to behavioral abnormalities [37]. We used the following tests: Mini Mental State Examination for general cognitive status - Rey Auditory Verbal Learning Test and Digit Span forward, two efficient neuropsychological instruments for testing verbal memory – Immediate Visual Memory Test (an Italian visuospatial test) and Corsi Block-Tapping Test forward, used to measure visuospatial memory - Copying of Drawings with and without elements of programmation, two common tools to evaluate visuospatial abilities - Attentive Matrices for both selective and sustained attention - Raven's Progressive Matrices, a widely used non verbal intelligence test for abstract reasoning - Digit Span backward, Corsi Block-Tapping Test backward, Phonological Verbal Fluency Test, Trail Making Test A, Trail Making Test B, Wisconsin Card Sorting Test, Analogies Test and Time and Weight Estimation Test, STEP, to investigate deeply the presence of executive dysfunctions [38]–[42].

Unadjusted analysis was performed as previously described [26]. Briefly, for each patient, the raw scores from each test were compared with published norms (age-, sex-, and education level-corrected, when necessary) and transformed into Z scores to express the deviation from the normal mean [Z = (raw data−test mean)/test standard deviation] [26]. Mean domain Z scores (MDZs) were defined as the average of the Z scores from the tests comprising each domain. To indicate cognitive function as a composite score, the Z score for each domain was transformed into a Domain Cognitive Dysfunction score (DCDs), with higher values representing more impairment in a particular domain. The sum of all DCDs across the 5 domains resulted in the Global Cognitive Dysfunction score (GCDs), which was transformed into a Global Cognitive Dysfunction category (GCDc) (Table 1).

**Table 1:** Scoring and categorization of cognitive dysfunction*.

**Test raw scores**	Obtained from performance on the neurocognitive testing
**Test Z scores**	Compared with age- and sex-matched published normal values
**Mean Domain Z scores (MDZs)**	Average of the Z scores in the tests comprising each domain
**Domain Cognitive Dysfunction Score (DCDs)**	1) if MDZs≥−1, then DCDs = 0;2) if −2≤MDZs<−1, then DCDs = 1;3) if MDZs<−2, then DCDs = 2;
**Global Cognitive Dysfunction Score**	Sum of Domain Cognitive Dysfunction Scores over the 5 domains (max 10)
**Global Cognitive Category**	Defined from Global Cognitive Dysfunction Score (GCDs)
**Absent**	GCDs 0–1
**Mild**	GCDs 2–3
**Moderate**	GCDs 4–5
**Severe**	GCDs≥6

* The composite score is constructed from the bottom to the top of the table.

The statistical calculations were performed using Statistical Package for Social Sciences 13.0 (SPSS, Chicago, IL, USA) and GraphPad 5.0 (La Jolla, CA, USA). Normally distributed variables were summarized using the mean ± SD, and non-normally distributed variables by the median and range. Wilcoxon's matched pairs test and paired t-test were performed. Univariate comparisons between nominal variables were calculated using chi-square (χ^2^) test or Fisher-test where appropriate. Two-tailed P values were reported, P values less than or equal to 0.05 were considered significant.

GCDs were compared in patients grouped by antibody level. The binary outcomes variable for the antibody testing were serum autoantibody status, defined either as present versus absent or low/absent versus high. The results were verified through analysis of the domain Z scores and single-test Z scores. Descriptive statistics were computed for all study variables. Multivariable logistic regression analysis was performed to explore any effect of anti-cardiolipin IgM, sex, age, educational level, SLEDAI, SLICC, and corticosteroid dosage on GCD. In this regression, only variables that achieved P value<0.100 in the univariate analysis were included for calculation.

## Results

Clinical and demographic features of the patients are shown in Table S1. All the patients, were Caucasian and showed a relatively high level of education (mean 12.1 years), thus an expected relatively high level of cognitive function. At the time of study entry, mean daily prednisone dosage was relatively low and a notable percentage (22.4%) was taking no steroids, in accordance with the high number of patients with low disease activity or with complete remission (Table S1).

**Figure 1:**
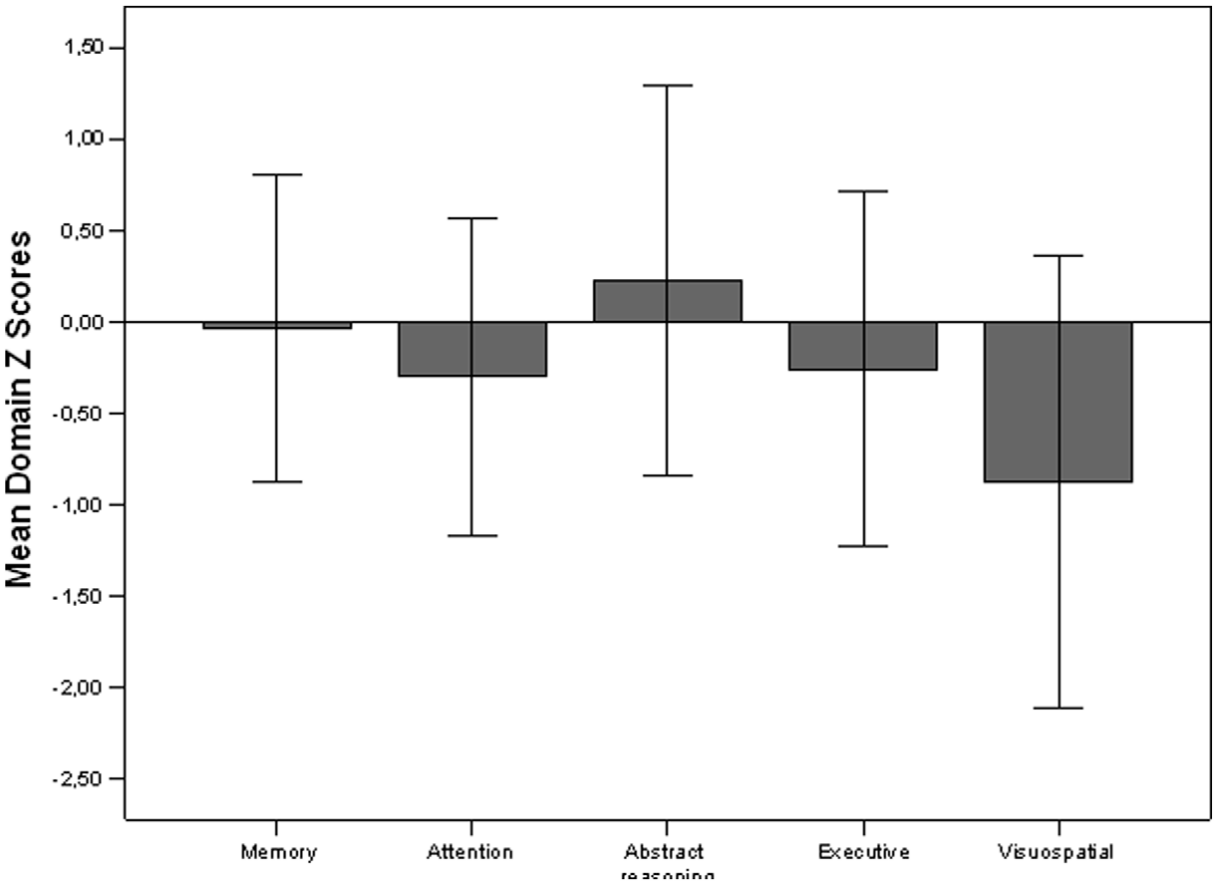
Distribution of neurocognitive impairment, expressed as MDZ scores±SD, in the patients enrolled (Memory MDZ −0.03±0.84, attention MDZ −0.3±0.87, abstract reasoning 0.23±1.1, executive MDZ −0.26±0.97, visuospatial MDZ −0.87±1.24).

When considering the patients' mean domain Z scores, visuospatial domain was the most compromised (MDZs = −0.89±1.23), and it was the only test in the mildly impaired range (Z score of −0.6 or less), as shown in Figure 1.

After transforming the MDZs into DCDs (Table 1), the percentage of patients with impairment in the diverse domains was calculated as shown in Table 2. Again, the visuospatial domain showed the highest percentages of patients with some CI. Considering the GCDs, 11 patients (19%) had mild GCDs impairment (GCDs 2–3), 4 patients (7%) showed moderate impairment (GCDs 4–5) and 3 patients (5%) had severe dysfunction (GCDs≥6). Fourteen patients (24.1%) showed pathological MMPI. However, when comparing patients with MMPI impairment and those without, there were no significant differences in GCDs (2.3±2.7 vs 1.1±1.5, respectively, P = 0.868).

**Table 2:** Percentage of patients with neurocognitive impairment, expressed as DCDs, in the diverse domains considered.

	Memory	Attention	Abstract reasoning	Executive	Visuospatial
***DCDs*** ** = 0**	90%	82%	85%	83%	58%
***DCDs*** ** = 1**	5%	15%	10%	10%	27%
***DCDs*** ** = 2**	5%	3%	5%	7%	15%

DCDs: Domain Cognitive Dysfunction score.

Anti-CL IgM were present in 6 (10.3%) patients, anti-CL IgG in 7 (12.1%), anti-β2GPI IgM in 6 (10.3%), anti-β2GPI IgG in 7 (12.1%), anti-P ribosomal in 12 patients (20.2%), AECA in 6 patients (10.3%), and anti-Nedd5 in 15 (28.8%).

Within the autoantibodies tested, aCL IgM were associated with impairment in the visuospatial domain (χ^2^ = 8.658; P = 0.013).

Univariate analysis of the correlation between anti-phospholipid antibodies with cognitive function is reported in Table 3.

**Table 3:** Univariate analysis of the correlation between anti-phospholipids antibodies in SLE patients with neurocognitive assessment.

Neurocognitive assessment	Paerson's Significance	anti-CL IgM (MPL)	anti-CL IgG (GPL)	anti-β2IgM (MPL)	anti-β2IgG (GPL)
*Memory MDZs*	r	0.027	0.210	0.143	0.171
	P value	0.847	0.139	0.311	0.231
*Attentional MDZs*	r	0.050	**0.348**	0.214	0.230
	P value	0.725	**0.012**	0.128	0.104
*Abstract reasoning MDZs*	r	−0.024	0.138	0.054	0.142
	P value	0.868	0.334	0.702	0.321
*Executive MDZs*	r	0.068	**0.371**	0.164	**0.341**
	P value	0.634	**0.007**	0.246	**0.014**
*Visuospatial MDZs*	r	−0.148	0.182	0.009	0.220
	P value	0.294	0.201	0.948	0.121
*Memory DCDs*	r	−0.095	−0.056	−0.088	−0.055
	P value	0.502	0.697	0.537	0.702
*Attentional DCDs*	r	−0.010	−0.094	−0.088	−0.093
	P value	0.942	0.510	0.535	0.518
*Abstract reasoning DCDs*	r	0.033	−0.082	−0.053	−0.081
	P value	0.817	0.567	0.709	0.574
*Executive DCDs*	r	0.010	−0.082	−0.021	−0.081
	P value	0.943	0.567	0.880	0.574
*Visuospatial DCDs*	r	**0.283**	−0.155	0.065	−0.152
	P value	**0.042**	0.278	0.648	0.287
*GCDs*	R	0.101	−0.143	−0.037	−0.140
	P value	0.475	0.317	0.793	0.326

MDZs: Mean Domain Z score; DCDs: Domain Cognitive Dysfunction score; GCDs: global cognitive dysfunction score.

No further association, neither correlation, was found with the other autoantibody tested (anti-dsDNA, anti-P ribosomal, AECA, anti-Nedd5).

Univariate analysis of the correlation between educational level, SLEDAI and SLICC with cognitive function is reported in Table 4.

**Table 4:** Univariate analysis of the correlation between education level, SLEDAI and SLICC in SLE patients with neurocognitive assessment.

Neurocognitive assessment	Paerson's Significance	Education level	SLEDAI	SLICC
*Memory MDZs*	R	−0.108	−0.266	−0.236
	P value	0.421	0.052	0.077
*Attentional MDZs*	R	−0.025	**−0.479**	**−0.304**
	P value	0.853	**0.000**	**0.022**
*Abstract reasoning MDZs*	R	−0.224	−0.007	−0.099
	P value	0.091	0.960	0.462
*Executive MDZs*	R	−0.136	−0.252	−0.012
	P value	0.309	0.066	0.931
*Visuospatial MDZs*	R	**−0.334**	−0.150	**−0.271**
	P value	**0.010**	0.280	**0.041**
*Memory DCD*	R	0.156	0.215	0.191
	P value	0.243	0.119	0.154
*Attentional DCD*	R	−0.001	**0.420**	0.086
	P value	0.993	**0.002**	0.527
*Abstract reasoning DCD*	R	**0.323**	−0.080	0.177
	P value	**0.013**	0.566	0.187
*Executive DCD*	R	0.086	**0.330**	0.061
	P value	0.523	**0.015**	0.651
*Visuospatial DCD*	R	**0.347**	0.139	**0.301**
	P value	**0.008**	0.317	**0.023**
*GCD*	R	**0.285**	**0.283**	**0.283**
	P value	**0.030**	**0.038**	**0.038**

MDZs: Mean Domain Z score; DCDs: Domain Cognitive Dysfunction score; GCDs: global cognitive dysfunction score.

No further correlation was found with the other clinical features tested (age, sex, disease duration, prednisone dosage).

The statistical significance of such correlations remained also when adjusted for age, sex, and steroid therapy. In the logistic regression analysis, GCD was inversely associated with the education level (P = 0.025), while no effect was found for anti-cardiolipin IgM, sex, age, SLEDAI, SLICC, and corticosteroid dosage.

## Discussion

Cognitive dysfunction is common in patients with SLE, and several studies pointed out the relevance of such problem on patients' health-related quality of life, functional outcome, and employment [38]. We found CI in a relevant percentage of Italian SLE patients, with an overall prevalence of 31% (19% mild, 7% moderate and 5% severe), a prevalence in the average when compared with results from previous studies that ranged between 6% and 79% [4], [10]–[14], [26]. Indeed, the prevalence of CI greatly varied between different cohorts. Sanna et al. and Hanly et al. reported the lowest percentages (11% and 6%, respectively) [11], [43]; however, their results could have been affected by the retrospective study design. Nonetheless, results from our cohort are lower than that from other Italian populations. Afeltra et al. reported in 2003 a prevalence of 52% of CI, which was significantly associated with higher levels of anti-PL antibodies [12]. Probably, our results may reflect the high education level and the low levels of disease activity observed in our cohort. The visuospatial domain was the most affected in the present study, suggesting a fronto-parietal deficit. In addition, any level of CI was observed across the 5 domains in a notable percentage of patients. The presence of behavioral abnormalities in a relevant percentage of our patients (24%) evaluated with MMPI test, did not alter the significance of our results. Indeed, these patients showed mean GCDs levels similar to those without MMPI impairment.

According to previous reports [44], we found an association between anti-PL and cognitive dysfunction, specifically visuospatial domain, in SLE patients. Furthermore, education, disease activity and chronic damage positively correlated with impairment in cognitive functions.

Since the first report from Hanly et al., the role of anti-CL antibodies in CI has been explored leading to conflicting results [44]. In 1995 Schmidt et al. found that increased aCL titers in normal elderly persons may be associated with subtle neuropsychological dysfunction [45]. Again, Hanly et al. suggested that anti-CL IgG and IgA may be responsible for long-term subtle deterioration in cognitive function in patients with SLE [13]. Anti-PL may provoke CNS damage by several mechanisms, for example, by modulating neuronal functions or by directly causing thrombosis within vessels of minute caliber [46]. Moreover, anti-PL may contribute to neurological damage by reacting with brain cells (astrocytes, neuronal cells, and endothelial cells) by means of β_2_GPI interactions as previously demonstrated by our group, suggesting that these cells may represent the autoantibody target [47]–[49]. Shoenfeld et al. administered intracerebro-ventricularly (ICV) immunoglobulins (IgG) from patients with APS to mice, demonstrating a direct binding to neuronal structures in the hippocampus and cerebral cortex. Furthermore, these mice injected with IgG performed worse in the water maze compared to the controls [50]. It was also showed that when female BALB/c mice were immunized once with β2-GPI in complete Freund's adjuvant (CFA) or with CFA alone (controls), the APS mice develop elevated levels of antibodies against negatively charged phospholipids and β2-GPI, accompanied by neurological impairment consisting of both cognitive and behavioral changes [51].

Herein, we demonstrated the association between cognitive dysfunction and anti-CL in a cohort of patients without a history of cerebral thromboses. This evidence may suggest that anti-CL may have worked by modulating neuronal function rather than directly provoking thrombosis. Interestingly, the results showed that the visuospatial domain impairment was also correlated with serum anti-CL IgM titers, suggesting a potential pathogenic role of these antibodies in brain areas related with working-memory.

The other autoantibodies tested were not found associated with CI. Concerning anti-dsDNA and anti-P ribosomal, we can consider our result as a confirmation of previous studies [20], [52], [53]. Furthermore, for the first time we studied whether AECA and anti-Nedd5 C-ter were associated with cognitive dysfunction in SLE. These antibodies have been previously associated with other manifestations of NPSLE [24], [25], [54], and a relationship was found between AECA and anti-Nedd5 C-ter with psychosis and depression in SLE patients [24], [25]. In our cohort, no association was found between these autoantibodies and CI. It is interesting to note that anti-P ribosomal antibodies, which have been mostly associated with NPSLE psychosis [53], were not associated with CI in the present study.

It is not surprising that cognitive deficiency is associated with lower education level, disease activity and chronic damage in NPSLE patients. Poorer education *per se* may account for lower scores in neurocognitive test batteries [55]. The association between disease activity and CI is still uncertain. Carbotte et al. did not demonstrate this association in an investigation performed in 1995 [56]. However, this study had some limitations: the disease activity was assessed using the Lupus Activity Criteria Count in all patients while the SLEDAI was used only in a subset of twenty of them, and a relevant percentage of these patients also had CNS involvement at the time of the study. In contrast with Carbotte's results and similar to ours, Mikdashi & Handwerger in 2004 showed that higher disease activity, assessed by SLEDAI, may be an independent predictor of CI in SLE patients [19]. As mentioned above, our patients had an overall low disease activity (SLEDAI≤4 in 86.2%), in a percentage similar to that from other studies. Interestingly, our patients with active disease (SLEDAI>4) showed poorer performance in specific areas (attention and executive domains) than the group with inactive disease at the time of testing. Therefore, it seems that cognitive dysfunction in SLE patients may reflect an immune-mediated compromise of an underlying neuronal substrate possibly impaired by some non-specific effects of active illness affecting specific neurocognitive domains.

One of the concerns in testing CI in SLE patients is the possibility that symptoms are often fluctuating and sometimes evanescent. Furthermore, the assessment of CI has often not been exhaustive in early studies. Only recently the ACR has proposed a brief but standardized research battery [1]. We used an extensive battery of tests that deeply explore cognitive functions and allow for the proper selection of the relevant factors involved in the development of CI in our NPSLE patients. Our study design used an *a priori* grouping of test batteries to reflect dysfunction in different cognitive domains, employed a system in which domain Z scores were transformed into the DCDs and further into the composite score - the GCDs. This approach enabled us to compare patients with severe abnormalities in one or two domains with patients showing mild or moderate changes in several domains. We adopted and successfully reproduced this method from Lapteva *et* al, who consistently used this approach and showed its validity [26].

In conclusion, anti-PL, disease activity, chronic damage and educational level are associated with cognitive dysfunction in SLE. The use of a wide spectrum of tests allowed for a better selection of the relevant factors involved in SLE cognitive dysfunction, and standardized neuropsychological testing methods should be used for routine assessment of SLE patients.

## Supporting Information

Table S1 Demographic and clinical characteristics of SLE patients.
(DOC)
