# Supplementary material for: Neurocognitive Dysfunction in Systemic Lupus Erythematosus: Association with Antiphospholipid Antibodies, Disease Activity and Chronic Damage
Source: PLoS One. 2012 Mar 26;7(3):e33824. doi: 10.1371/journal.pone.0033824 (PMC3312889; doi:10.1371/journal.pone.0033824)
Supplement: Table S1 — Demographic and clinical characteristics of SLE patients. (DOC) [file pone.0033824.s001.doc]

**TABLE S1.** Demographic and clinical characteristics of SLE patients.

| **Characteristic** | Patients (N=58) |
| --- | --- |
| M/F | 7/51 |
| Age (years) mean±SD (range) | 38.1±12.6 (16-71) |
| Disease duration (months) mean±SD (range) | 112.1±77.9 (7-284) |
| History of systemic involvement |  |
| Renal disorder N(%) | 21 (36.2) |
| Serositis N(%) | 19 (32.8) |
| Cytopenia N(%) | 47 (81.0) |
| Arthritis N(%) | 40 (69.0) |
| NPSLE N(%) | 15 (25.9) |
| Headache N(%) | 8 (53.3) |
| Anxiety disorders/Mood disorders N(%) | 7 (46.7) |
| Seizure disorders N(%) | 6 (40) |
| Cognitive dysfunction N(%) | 4 (26.7) |
| Polyneuropathy N(%) | 2 (13.3) |
| Mucocutaneous N(%) | 53 (91.4) |
| malar rash N(%) | 38 (65.5) |
| discoid rash N(%) | 7 (12.1) |
| oral ulcer N(%) | 18 (31.1) |
| photosensitivity N(%)  Anti-phospholipid Syndrome | 24 (41.4)  8 (13.7) |
| Immunologic abnormalities* (besides ANA) N(%) | 41 (70.7) |
| Current daily prednisone dosage (mg/day) mean±SD | 9.9±8.9 |
| 0 mg/day N(%)  ≤5 mg/day N(%)  >5–≤10 mg/day N(%)  >10–≤20 mg N(%)  ≥20 mg/day N(%) | 13 (22.4) |
|  | 9 (15.5) |
|  | 14 (24.1) |
|  | 12 (20.7) |
|  | 10 (17.2) |
| Cumulative prednisone dosage |  |
| ≤10 gm N(%)  10–20 gm N(%)  ≥20 gm N(%) | 20 (34.5) |
|  | 10 (17.2) |
|  | 28 (48.3) |
| Immunosuppressants |  |
| Hydroxychloroquine N(%) | 40 (69.0) |
| Mycophenolate mofetil N(%) | 12 (20.7) |
| Cyclophosphamide N(%) | 2 (3.4) |
| Methotrexate N(%) | 4 (6.9) |
| Cyclosporine A N(%) | 3 (5.2) |
| Azathioprine N(%)  Anti-coagulants N/(%)  Low Dose Aspirin | 5 (8.6)  5 (8.6)  12 (20.7) |
| SLEDAI mean±SD | 1.6±1.7 |
| SLEDAI ≤ 4 N(%) | 50 (86.2) |
| SLICC mean±SD | 2.8±4.3 |
| Education (years) mean±SD | 12.1±3.5 |

SD: Standard Deviation; NP: NeuroPsychiatric; ANA: Anti-Nuclear Antibody; SLEDAI: Systemic Lupus Erythematosus Disease Activity Index; SLICC: Systemic Lupus International Collaborating Clinics.

*As stated in 1997 ACR Classification criteria for SLE (25)
